# Long non-coding RNA in stem cell pluripotency and lineage commitment: functions and evolutionary conservation

**DOI:** 10.1007/s00018-018-3000-z

**Published:** 2019-01-03

**Authors:** Annalisa Fico, Alessandro Fiorenzano, Emilia Pascale, Eduardo Jorge Patriarca, Gabriella Minchiotti

**Affiliations:** 1 Stem Cell Fate Laboratory, Institute of Genetics and Biophysics “A. Buzzati-Traverso”, CNR, 80131 Naples, Italy; 2 Institute of Genetics and Biophysics “A. Buzzati-Traverso”, CNR, 80131 Naples, Italy; 3 Developmental and Regenerative Neurobiology, Wallenberg Neuroscience Center, and Lund Stem Cell Centre, Department of Experimental Medical Science, Lund University, 22184 Lund, Sweden

**Keywords:** Embryonic stem cells, LncRNAs, Ultraconserved elements, T-UCEs

## Abstract

LncRNAs have recently emerged as new and fundamental transcriptional and post-transcriptional regulators acting at multiple levels of gene expression. Indeed, lncRNAs participate in a wide variety of stem cell and developmental processes, acting in *cis* and/or in *trans* in the nuclear and/or in the cytoplasmic compartments, and generating an intricate network of interactions with RNAs, enhancers, and chromatin-modifier complexes. Given the versatility of these molecules to operate in different subcellular compartments, via different modes of action and with different target specificity, the interest in this research field is rapidly growing. Here, we review recent progress in defining the functional role of lncRNAs in stem cell biology with a specific focus on the underlying mechanisms. We also discuss recent findings on a new family of evolutionary conserved lncRNAs transcribed from ultraconserved elements, which show perfect conservation between human, mouse, and rat genomes, and that are emerging as new player in this complex scenario.

## Introduction

Most of the mammalian genome (> 90%) is transcribed into non-coding RNA (ncRNA), once controversially known as ‘junk DNA’ because of its inability to encode proteins and the absence of evolutionary conservation [1–3]. In recent decades, however, large-scale genome-wide sequencing analysis has revealed the tissue-specific expression of ncRNAs and their functional importance as essential regulators in fundamental biological processes, dismantling the now obsolete paradigm of RNA as simply an intermediary between DNA and protein [4–6]. In this expanded view of both genomic and transcriptomic analysis, thousands of long ncRNAs (lncRNAs) have been identified and classified to include any transcript with primary sequence longer than 200 nucleotides [7]. Based on this arbitrary cut-off, lncRNAs are distinct from more extensively studied classes of short ncRNA, such as transfer RNA, microRNA (miRNA), and small nucleolar RNA [8]. LncRNAs share several features with coding mRNAs (both are transcribed by RNA polymerase II and further capped and spliced [9]), but have lower expression levels, are longer in length, and are involved in many different regulatory circuitries, reflecting their multifunctional role in cells [10]. For instance, they can fold into complex three-dimensional structures able to bind DNA, RNA, and protein molecules, thus determining complex regulatory networks in both the cytoplasm and nucleus [11]. Through distinct modes of action, they are also able to (1) regulate chromatin state and methylation, recruiting remodeling factors in *cis* or in *trans*, (2) act as scaffolds for interactions between proteins by tethering them to complexes that enable transcription factors (TFs) and recruit chromatin modifiers, (3) impact on genome targeting by serving as guides, (4) function as decoys (also referred to as sponges) for miRNA target sites able to sequester and inactivate miRNA function, and (5) mediate antisense interference for coding mRNA [12]. Very recent studies investigating these particular characteristics showed that lncRNAs are essential to establish developmental patterning and maintain the pluripotency network, further underscoring their important role in stem cell biology/technology, and in particular cellular reprogramming [13, 14]. As the majority of loci transcribed into lncRNA display low expression levels and poor conservation in the other species, the question of how many human lncRNAs are actually functional is still debated. However, about 1000 human lncRNAs show moderate-to-high expression as well as signs of evolutionary constraint, and around 300 of these are conserved in other non-mammalian vertebrates [15]. Increasing interest is emerging on lncRNAs in embryonic stem cell biology [16–18]. Here, we present an overview of current knowledge on the functional mechanisms of lncRNA in stem cell pluripotency and lineage commitment, highlighting the importance of evolutionary conservation in this context. We also focus on a new family of evolutionary conserved lncRNAs transcribed from ultraconserved elements (UCEs) known as *Transcribed UCEs* (T-UCEs), sequences of DNA which exhibit the unique feature of retaining extended perfect sequence identity between human, mouse, and rat genomes. This high level of conservation suggests a significant role for T-UCEs during embryogenesis and in stem cell lineage commitment, introducing a novel layer of biological regulation and determining new candidate targets for stem cell-based therapy and other clinical applications.

## Mode of action and cellular localization of lncRNAs

Although lncRNAs are reported to be involved in several processes related to physiology and/or disease, only a few have been functionally and mechanistically characterized [11]. The intracellular localization of lncRNAs is normally predictive of their mode of action [11].

**Fig. 1:**
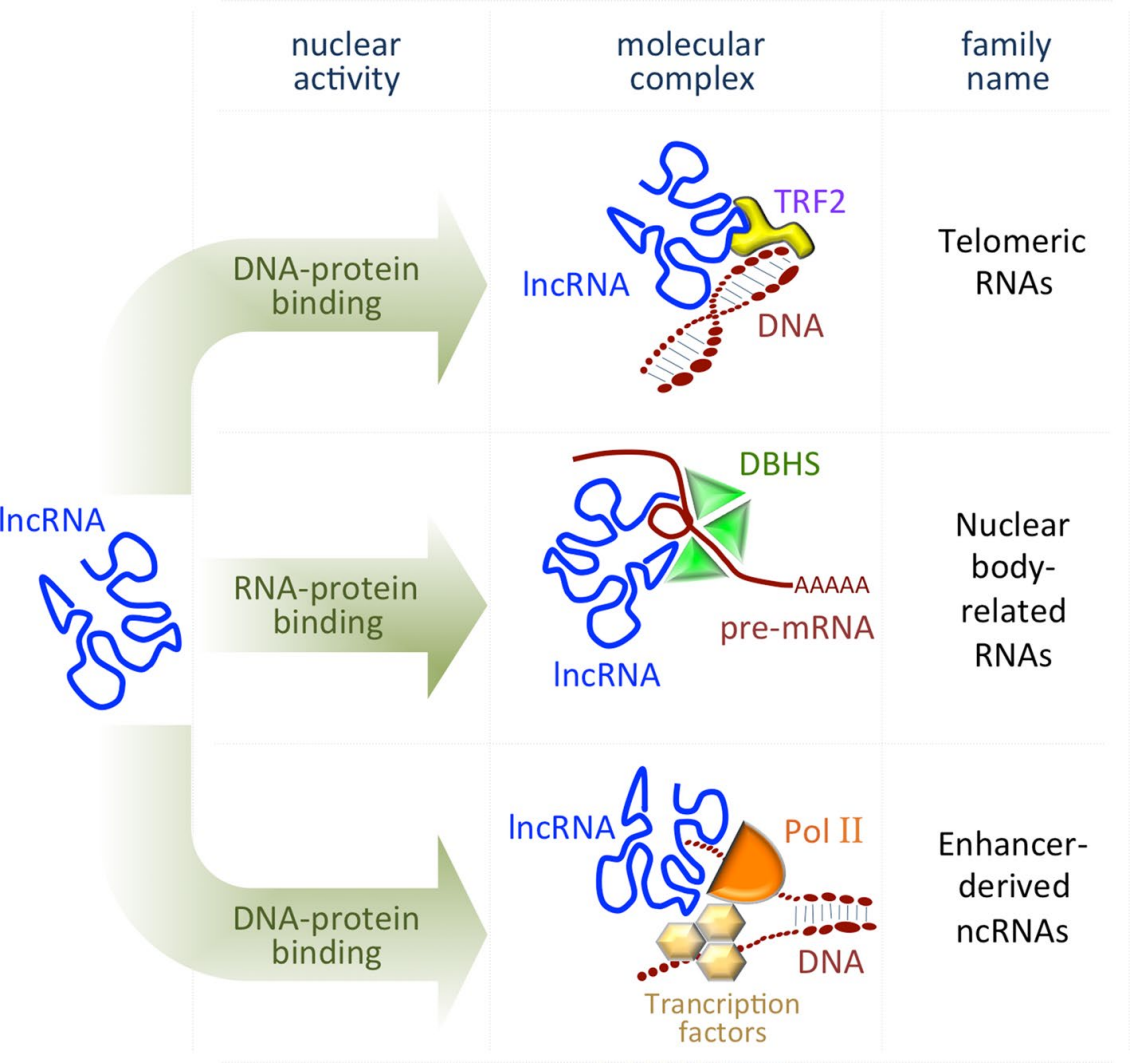
Scheme representing the main mode of action of the lncRNAs localized in the nucleus. *TRF2* telomeric repeat-binding factor 2, *DBHS* drosophila behavior human splicing proteins

In general, nuclear lncRNAs guide chromatin-modifying complexes to precise genomic loci and/or act as molecular scaffolds connecting distinct, but functionally related proteins [19]. As they are able to interact with other nucleic acids forming DNA/RNA duplexes, lncRNAs can exert either repressive or promoting activities on target genes by coordinating protein and RNA interactions, both in *cis* (on neighboring genes) and in *trans* (on distant loci) [20–22] (Fig. 1).

*Enhancer*-*derived ncRNAs* (eRNAs), a group of *cis*-acting lncRNAs, are functional transcripts associated with active enhancer sequences involved in many gene activation programs. Specifically, they play a fundamental role in guiding chromatin-remodeling complexes to specific promoters and in mediating chromatin loop formation [23]. A recent report identified an eRNA, transcribed at distal super-enhancer 45 kb upstream of the *Nanog* locus, which regulates the two nearest neighbor genes *Nanog* and *Dppa3*, two essential embryonic stem cell (ESC) pluripotency core factors [24]. Emerging evidence indicates that eRNAs are involved in ESC patterning and during differentiation. Using an integrated epigenomic screening approach, a set of eRNAs expressed during cardiac differentiation of ESCs was identified [25]. The expression of these transcripts correlates with the expression of their target genes, located in genomic proximity, which are robustly downregulated after genetic depletion of eRNAs. Intriguingly, eRNAs exhibit distinct expression dynamics, being inhibited in a negative regulatory loop when target mRNAs reach maximal intracellular levels or physiologically increased during stress response in adult heart. Overall, these data highlight a functional role for cardiac eRNAs in heart development and cardiac remodeling after injury. *MesEndoderm Transcriptional Enhancer Organizing Region* (*Meteor*), another eRNA specifically expressed in ESCs, is indispensable during mesendoderm specification and subsequently cardiac differentiation, supporting the involvement of this class of genomic elements in ESC specification during development [26].

*Divergent* lncRNAs are another group of *cis*-acting lncRNAs that are transcribed in the opposite direction to nearby coding genes [27]. By way of an example, the divergent lncRNA *Evx1as* regulates transcription of its neighbor gene *EVX1*, enhancing ESC mesendoderm differentiation. Mechanistically, *Evx1as* binds to chromatin regulatory sites and, through interaction with Mediator, a multiprotein complex that functions as a transcriptional co-activator, establishes an active chromatin state [28].

Many other lncRNAs act at a distance, regulating gene expression in *trans* via tethering specific protein partners. *Pnky*, an evolutionarily conserved neural-specific transcript, controls mouse and human neurogenesis by preserving neural stem cells in embryonic and post-natal brain. *Pnky* specifically interacts with polypyrimidine tract-binding protein 1, an RNA-splicing factor that is a potent regulator of neural development [29], and this complex regulates a set of transcripts associated with neuronal differentiation. Phenotypically, *Pnky* downregulation promotes neuronal differentiation by increasing the number of cell divisions and depleting the pool of neural progenitors [30].

**Fig. 2:**
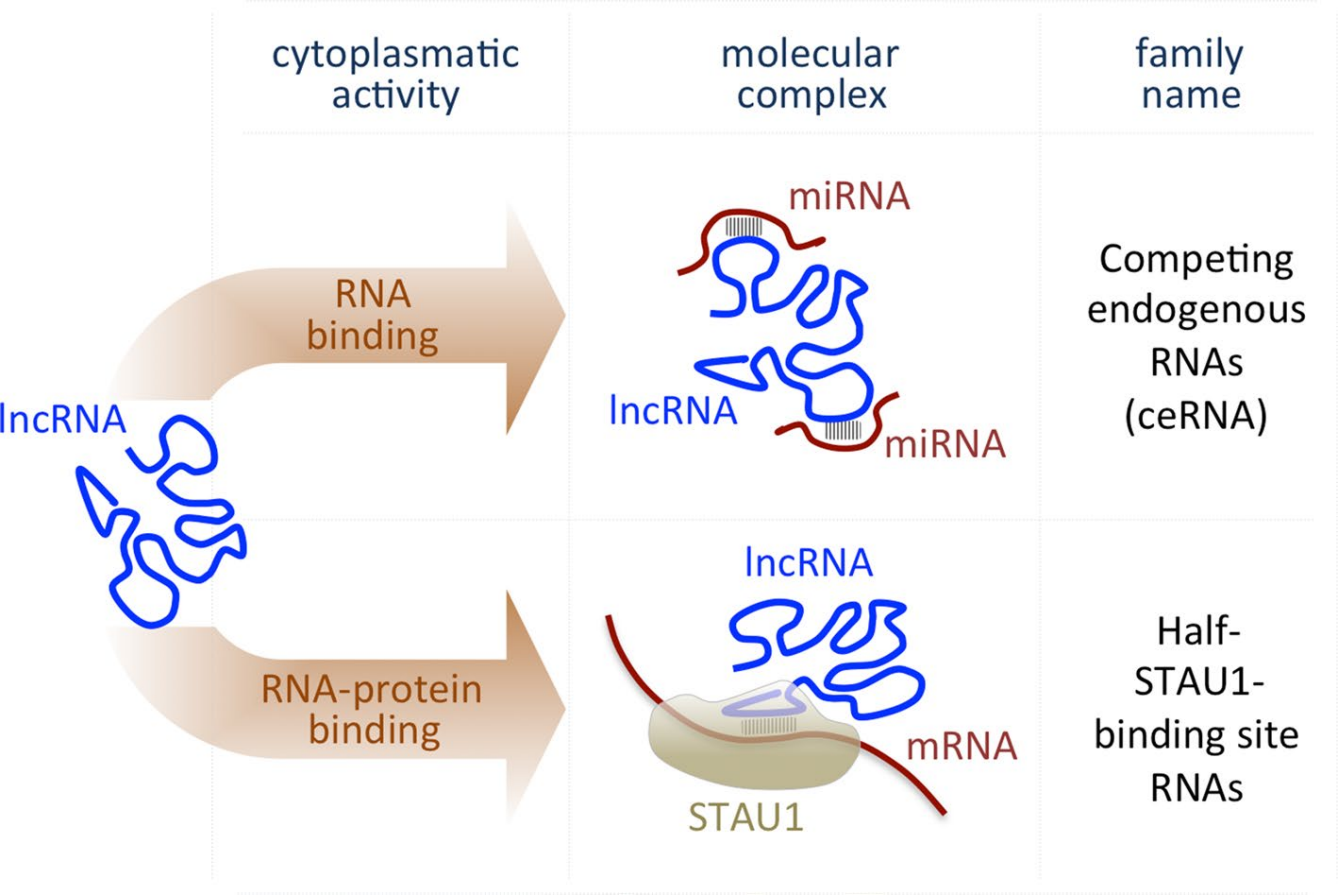
Scheme representing the main mode of action of the lncRNAs localized in the cytoplasm. *STAU1* Staufen 1 protein

Although the shuttling mechanisms remain unclear, a large fraction of lncRNAs is exported to the cytoplasm, where they act as important post-transcriptional regulators. As a result of their ability to bind RNA targets through complementary base pairing, lncRNAs regulate gene expression via mRNA degradation or by mediating translational repression (Fig. 2). Cytoplasmic lncRNAs include *Competing Endogenous RNAs* (ceRNAs), which can indirectly enhance protein translation by sequestering miRNAs that would, otherwise, inhibit downstream target mRNAs. This mechanism was shown to be involved in differentiation and several cancer types [31–37]. Circular RNAs are a cryptic class of sponging lncRNAs [38, 39], whose peculiar circular structure provides greater stability than other transcripts. Finally, in humans, several cytoplasmic lncRNAs transactivate Staufen1-mediated mRNA decay by duplexing with 3′-UTRs via Alu elements [40, 41]. However, little is known of the specific molecular functions of these transcripts (Table 1).

**Table 1:** Subcellular localization and function of lncRNAs

Cellular localization	Group/family	*Cis*- and *trans*-activity	Examples	References
Nuclear	Enhancer-derived ncRNAs (eRNA)	*Cis*	*Meteor*	[42]
	Divergent lncRNAs	*Cis*	*Evx1as*	[28]
	Pseudogenes	*Trans*	*Oct4P4*	[43]
	Telomeric RNAs	*Trans*	TERRA	[44]
	Nuclear body-related lncRNAs	*Cis*/*Trans*	*Neat1*	[45, 46]
Cytoplasmatic	Competing endogenous RNAs (ceRNAs)	*Trans*	CircularRNA	[38, 39]
	Half-STAU1-binding site RNAs	*Trans*	AF087999	[40, 41]

## LncRNAs are key regulators of pluripotency and differentiation

### LncRNAs as members of the pluripotency core network

Several studies identified lncRNAs as fundamental regulators of molecular mechanisms orchestrating the intricate gene regulatory network that controls ESC pluripotency and cell lineage determination [20, 47–51]. In a large-scale functional study, individual knockdown of more than 130 lncRNAs was shown to cause a clear perturbation of the transcriptome, often leading to ESC pluripotency loss [52]. Most lncRNAs involved in mESC self-renewal are transcriptionally regulated by pluripotency core TFs Oct4, Nanog, and Sox2. *Tcl1 Upstream Neuron*-*Associated* lincRNA (*TUNA*, also known as *megamind*) is a very well-characterized lncRNA required for mESC proliferation and maintenance of self-renewal [53]. TUNA forms an RNA–multiprotein complex, which activates transcription of *Nanog* and *Sox2* upon binding to their promoters. Consistent with its expression in neural progenitors and zebrafish, mouse, and human central nervous system (CNS), TUNA depletion also dramatically impairs neural lineage commitment [54]. Further examples of lncRNAs involved in self-renewal include *AK028326* (Oct4-activated) and *AK141205* (Nanog-repressed), direct targets of OCT4 and NANOG, respectively. Knockdown and overexpression of these transcripts lead to substantial changes in *Oct4* and *Nanog* mRNA levels, with consequent alterations in mESC pluripotency and cellular lineage-specific gene expression [55]. The interplay between pluripotency core TFs and lncRNAs has also been described in hESCs. For example, the lncRNAs *ES1*, *ES2*, and *ES3* were found new regulators of pluripotency and neurogenesis through interaction with SOX2 [51].

*Linc*-*RoR* (*Long intergenic non*-*protein-coding RNA*, *Regulator of Reprogramming*) is well described as one of the few examples of cytoplasmic lncRNAs regulating pluripotency [47]. It was identified by Zhang and colleagues as an lncRNA able to promote cell reprogramming by inhibiting p53-mediated cell cycle arrest and apoptosis [56]. It was subsequently shown that *linc*-*RoR* also preserves hESC self-renewal by acting as a ceRNA. Specifically, in undifferentiated hESCs, *linc*-*RoR* sequesters mir-145 (able to inhibit translation of core TFs [57]) de-repress the translation of all pluripotency factors. Upon differentiation, *linc*-*RoR* expression is downregulated with consequent release of miR-145 and repression of *Oct4*, *Nanog,* and *Sox2* [58]. In addition, OCT4 activates *linc*-*RoR* and inhibits miR-145 at transcriptional level, underscoring the existence of a network between TFs, lncRNAs and, small RNAs that, in turn, fine-tunes ESC pluripotency/differentiation balance (reviewed by Rosa and Ballarino [16]).

### LncRNAs in chromatin modifications

Many nuclear lncRNAs interact with histone modifiers (writers, readers, and erasers) and/or other chromatin-associated proteins [52], thus acting as epigenetic regulators. A pivotal study using RNA immunoprecipitation sequencing identified several Polycomb repressive complex 2 (PRC2)-associated RNAs in ESCs at genome-wide level [59]. Although promiscuous RNA binding to the PRC2 complex is reported [60], the other studies highlight that lncRNA binding to this complex is important in modulating its interaction with cofactors that in turn confer its specificity of action. The lncRNA–PRC2 interaction involving *Maternally Expressed 3* (*Meg3*) and JARID2 in pluripotent stem cells is very well described. *Meg3* is a maternally expressed, imprinted lncRNA belonging to the *Dlk1*–*Dio3* gene cluster on chromosome 12qF1. Appropriate expression of these lncRNAs is required for embryonic development [61, 62] and to reach full pluripotency during cell reprogramming. Indeed, *induced Pluripotent Stem Cells* (iPSCs) carrying aberrantly silenced *Dlk1*–*Dio3* cluster genes are unable to contribute to development of chimeric mice and fail to pass tetraploid complementation assay [63]. JARID2 is a catalytically inactive Jumonji family histone demethylase that is essential for PRC2 recruitment in ESCs [64]. JARID2 is able to directly interact with about 100 lncRNAs in mESCs [65], including *Meg3*, which regulates the activity of PRC2 in ESCs by binding JARID2 [59]. Specifically, *Meg3* stabilizes PRC2 occupancy in *trans* at genomic loci encoding for factors involved in cell differentiation [59]. At a mechanistic level, *Meg3* acts as a scaffold to increase/stabilize the interaction between JARID2 and the PRC2 component EZH2, assembling the Polycomb complex on chromatin at specific JARID2 target sites. The specificity of this mechanism is achieved via RNA–DNA base pairing recognition between the lncRNA and the target gene [65].

Similarly, the lncRNA Braveheart (*Bvht*) directly binds PRC2. *Bvht* was shown to directly interact with SUZ12, a core component of the PRC2 complex, at numerous stages during ESC-cardiac differentiation. Interestingly, SUZ12 and its associated repressive modification H3K27me3 are enriched at the promoters of cardiac-associated genes such as *MesP1* in cells lacking *Bvht* expression. Notably, those genes remain bivalent in *Bvht*-depleted cells, similar to their initial configuration in ESCs, in line with the inability of these cells to trigger cardiac cell commitment. Indeed, *Bvht* has been described as a novel lncRNA that mediates specific cell commitment by epigenetic regulation of gene-expression programs [42].

Another well-characterized histone modifier that interacts with lncRNAs is WDR5, a component of mixed-lineage leukemia (MLL) complexes [66–68]. WDR5 is particularly important for mammalian ESC self-renewal and maintenance of active chromatin for pluripotency genes, and is required for efficient generation of iPSCs from differentiated somatic cells [69, 70]. In undifferentiated stem cells, it interacts with many lncRNAs [71] including *lincRNA*-*1592* and *lincRNA*-*1552*, which are involved in the maintenance of ESC pluripotency. In turn, WDR5 binds the promoters of these two lncRNAs, suggesting a *cis*-regulatory mechanism (detailed in the review by Rosa and Ballarino [16]).

A sense pseudogene–lncRNA-based mechanism of gene regulation at epigenetic level controlling cross-talk between pseudogenes and their ancestral genes is also described, in which the X-linked *Oct4* pseudogene controls mESC self-renewal [43]. Specifically, in differentiating mESCs, the lncRNA *Oct4P4* forms a complex with the histone-lysine *N*-methyltransferase SUV39H1, which translocates to the *Oct4* promoter silencing *Oct4* gene.

Among the lncRNAs functionally characterized, there is also *lncPress1*, identified by analyzing 40 lncRNAs highly expressed in undifferentiated hESCs and repressed during differentiation in a p53-dependent manner. The interaction between *lncPRESS1* and SIRT6, the main deacetylase of histone H3 on lysine 56 (H3K56ac), has been extensively studied in hESCs. Transcriptionally activated pluripotency genes show high H3K56ac levels, which decrease significantly during ESC differentiation [72, 73]. Mechanistically, *lncPRESS1* interacts with SIRT6 and blocks its chromatin localization by maintaining high levels of histone H3K56 acetylation at promoters of pluripotency genes, ensuring the hESC pluripotent state [74].

### LncRNAs in maintenance of nuclear architecture

The nuclear organization of hESCs is particularly compartmentalized, and nuclear organization remodeling is linked to epigenomic reprogramming during differentiation [75, 76]. LncRNAs, such as *nuclear*-*enriched autosomal transcript 1* (*Neat1*), are important components of paraspeckles and are required for the assembly and structural integrity of these nuclear bodies [45, 46, 77]. Undifferentiated hESCs lack *Neat1* expression and paraspeckles within the nucleus; interestingly, they both only appear upon differentiation, suggesting that lncRNAs maintaining nuclear structure integrity have a potential regulatory role in ESCs (detailed in the review by Ng and Stanton [50]).

### LncRNA in cell signaling pathways and metabolism

The importance of external stimuli and cell signaling in the tuning of ESC pluripotency/differentiation balance is extensively described [78–80]. In this scenario, lncRNAs are players in the molecular orchestra of the pluripotent regulatory network. Examples include the *Growth arrest*-*specific transcript 5* (*GAS5*), a known tumor suppressor and growth arrest-related lncRNA, which is highly expressed in hESCs and directly regulated by the pluripotency factors OCT4 and SOX2. Specifically, *GAS5* maintains TGFβ signaling by protecting TGFβ receptor family ligand NODAL expression from miRNA-mediated degradation, thereby promoting hESC and iPSC self-renewal and pluripotency [81, 82].

Another lncRNA involved in cell signaling pathways is the conserved transcript *Divergent to* Goosecoid (GSC) *Induced by TGFβ* family signaling (*DIGIT*). By mapping the genome-wide occupancy of SMAD3 during endoderm differentiation, *DIGIT* was identified as an lncRNA regulated by an enhancer bound by SMAD3 upon Activin stimulation. *DIGIT* controls definitive endoderm specification by positively regulating in *trans* the proximal mesendoderm regulator GSC, with which it is divergently transcribed. Depletion of the DIGIT transcript inhibits the induction of GSC during endoderm differentiation of both hESCs and mESCs [83]. *Telomeric RNA* (*TERRA* or *TelRNA*), another type of lncRNA, is also highly expressed in mESCs, but declines significantly upon differentiation, implying that it may be involved in the maintenance of cell pluripotency. Interestingly, *TERRA* is one of the targets of the Wnt/β-catenin signaling pathway and can mimic its self-renewal-promoting effect when overexpressed. *TERRA* was found to inhibit the transcription of TCF3, and this is likely its key contribution to maintenance of mESC self-renewal, as overexpression of TCF3 abolishes the self-renewal-promoting effect of *TERRA* [44].

Recent studies have also highlighted the involvement of lncRNAs in the regulation of metabolic pathways contributing to stem cell fate specification. For instance, the lncRNA Lncenc1 has been shown to preserve ESC self-renewal by regulating the transcription of glycolytic genes through interaction with two RNA binding proteins, PTBP1 and HNRNPK. Indeed, the absence of Lncenc1 leads to a significant reduction of glycolysis-associated genes expression, which eventually results in an impaired glycolytic activity [84].

## LncRNAs and the importance of evolutionary conservation

Evolutionary conservation is currently considered one of the most powerful and reliable parameters to identify functional sequences in the genome, highlighting their role as potential regulatory elements in key biological processes [85].

Although there are a few experimentally studied lncRNAs that are conserved at sequence level, most exhibit weak or imperceptible primary sequence conservation, reflecting a lack of evolutionary constraints. This opens to debate the functional importance of lncRNAs. However, similarities in canonical sequences cannot be used as the only criterion to measure evolutionary relatedness, as lack of conserved sequences does not imply per se lack of functional conservation; additional dimensions of conservation need to be considered for lncRNAs such as structure, function, and expression from syntenic loci [86]. For instance, the lncRNA *HOTAIR* shows a conserved function and genomic position in the *HOX*-*C* cluster, although it is barely conserved in primary sequence between human and mouse [87]. Similarly, *GAS5* is another good example of an lncRNA without substantial sequence conservation but unambiguous biological function [81, 82]. In addition, recent in silico studies provided greater insight into interspecies conservation based on secondary structures among lncRNA homologs, underscoring how structure is more important than primary sequence changes in lncRNA functionality (reviewed by Nitsche and Stadler [88]).

However, there are some examples of lncRNA showing some degree of conservation in their primary sequence. For example, *TUNA* is one of the best conserved lncRNAs associated with ESC biology. Discovered in zebrafish and named *megamind*, it is mainly involved in brain development but also expressed in spinal cord and eye tissue [54]. As previously mentioned, it also displays important functions in molecular mechanisms underpinning self-renewal and cell fate determination. The exonic regions of *TUNA* show untypical strong sequence conservation across vertebrates. In particular, it contains a sequence element of about 200 bp in length with more than 80% sequence similarity between human and zebrafish [54]. Such a level of conservation exceeds even that of most coding genes. Another possible exception is *TERRA*, which is conserved between human and yeast [8]. Well-studied functionally important lncRNAs with orthologs over a wide phylogenetic range of species include genes such as *DIGIT*, *Braveheart*, *Pnky,* and *Neat1* (Table 2).

**Table 2:** LncRNAs and their evolutionary conservation

lncRNA	Conservation	Function roles	References
*AK028326*	Poor conserved	Self-renewal	[8]
*AK141205*	Conserved	Self-renewal	[52]
*Braveheart*	Not conserved	Cardiovascular differentiation	[42]
*DIGIT*	Conserved	Meso-endoderm differentiation	[83]
*Evx1as*	Conserved	Mesoderm differentiation	[28]
*GAS5*	Poor conserved	Self-renewal	[81, 82]
*Hotair*	Poor conserved	Self-renewal Cell proliferation	[87]
*LincPRESS1*	Poor conserved	Pluripotency Cell cycle regulation	[74]
*LincRNA1592*-*1552*	Poor conserved	Pluripotency	[52]
*Lin*-*RoR*	Poor conserved	Pluripotency Self-renewal	[47]
*Meg3*	Conserved	Pluripotency Reprogramming	[62, 63]
*Meteor*	Conserved	Mesoderm specification	[26]
*Neat1*	Conserved	Differentiation	[77]
*Oct4P4*	Poor conserved	Self-renewal Cell proliferation	[43]
*Pnky*	Conserved	Neuronal differentiation	[30]
*TERRA*	Conserved	Pluripotency	[8]
*TUNA*	Conserved	Self-renewal Neural differentiation	[54]

The *Transcribed ultraconserved elements* (T-UCEs), known to be the class of lncRNAs with the highest level of evolutionary conservation, are of particular interest in this already complex scenario. In the following sub-sections, we provide an overview of the UCEs and a summary of major findings that support the importance of T-UCEs in controlling ESC and early embryonic lineage commitment, highlighting key molecular pathways, and discussing their potential significance for stem cell biology.

## Ultraconserved elements (UCEs): a close look at evolutionary conservation

Computationally identified for the first time by Bejerano and colleagues in 2004, UCEs are 481 genomic segments longer than 200 bp; they are rarely lost across a wide range of mammalian species, showing the signs of evolutionary sequence constraint [89]. UCEs display perfect conservation (100% identity with no insertions or deletions) between human, mouse, and rat, and also retain a high percentage of identity in chicken, dog, and fugu genome [90]. UCEs are found in all chromosomes except chromosome 21 and Y, and are commonly classified according to the most recent human genome assembly (hg20) into five subgroups: intergenic, intronic, exonic, partially exonic, and exon-containing [91]. Although their complete functional characterization is still a long way off, it is known that UCEs are not randomly distributed, and their position within the genome also seems to reflect their function. These constrained sequences are in fact mostly allocated in clusters, flanking, or embedding genes involved in important physiological processes, and act as splicing or enhancer factors [92]. Their extreme conservation could be due to the absence of annotated transposons near many UCEs during evolution [93]. It has also been observed that these transposon-free regions coincide with the so-called chromatin bivalent domains, which mark key regulatory genes in embryo development and ESC pluripotency, implying a potential correlation between UCEs and genome and chromatin architecture [94]. Extreme conservation over such long stretches of DNA indicates strong negative selection pressure, suggesting that UCE depletion may have a dramatic effect on mammalian development [95, 96]. Unexpectedly, the initial findings suggested that the deletion of some UCEs was dispensable for mouse viability, and that the phenotype was only detectable over many generations, raising the possibility that they may be phenotypically redundant [97, 98]. However, this apparent discrepancy was successfully resolved in a recent study by Dickel and colleagues. The authors clarified the role of ultraconserved sequences in the early development, re-establishing the intriguing hypothesis that extreme levels of UCE conservation mirror their functional importance in genomic regulation for the acquisition of cell identity [99]. Emerging evidence shows that a significant fraction of UCEs act both as gene-expression enhancer promoters and lncRNAs, postulating their dual function during mouse development. This new layer of regulation makes the biology of UCEs even more challenging and adds further complexity to their functional annotation, while reinforcing the concept that the extraordinary constraint on their sequence implies simultaneous multiple roles [100, 101].

## Ultraconserved enhancer

Ultraconserved loci are normally located near key developmental coding genes and can work as *cis*-acting regulatory genomic elements directing the expression of neighbor genes. By comparing next-generation sequencing data sets with readouts on the function of UCEs as enhancers in mouse embryonic development, it emerges that a large number of UCEs can be transcribed and act as enhancers concomitantly at specific developmental stages [102]. This indicates that transcription and enhancer functions overlap within the same DNA sequence. A genome-wide high-throughput in vivo screening study using transgenic mouse reporter assay systematically tested hundreds of ultraconserved DNA elements annotated at E11.5 [102]. Remarkably, on examining a single time point of mouse development, almost 50% were found to act as tissue-specific enhancers. Although it is still unclear whether UCEs are also able to enhance in *trans* expression of multiple genes located further away along a chromosome, these findings warrant in-depth exploration of UCEs as enhancers in the other stages of embryo development, including the earliest stages of embryogenesis when cell lineage segregation is established [99, 102].

Deletion of UC.248, UC.329, UC.467, and UC.482 in knockout (KO) mice was found to be compatible with life and development with no obvious deleterious defects, challenging the paradigm that in vivo depletions would lead to a lethal phenotype, thus pointing to a functional role for these non-coding ultraconserved sequences [98]. The hypothesis that extreme sequence constraint does not necessarily reflect key functions required for viability was only recently validated [99]. Using CRISPR/Cas9 genome editing, a series of KO mice was generated lacking individual or different combinations of seven UCEs (UC.463–UC.465 and UC.467–UC.470). These UCEs display transcriptional enhancer activity in telencephalon or diencephalon, and are located along the X chromosome within regions of DNA spanning the *Arx* gene encoding an important neuronal TF, whose mutation causes neurological and sexual development disorders. Although KO mice were still viable and fertile, in almost all cases every single deletion induced an aberrant phenotype with dramatic neurological and growth abnormalities, undermining the hypothesis of functional redundancy [99]. Moreover, pair-wise losses in various combinations of these seven UCEs increased the severity of the mutant phenotype. After post-natal mouse brain dissection, the rodents showed abnormal alterations of neuron populations and substantial structural brain defects accompanied by decreasing fitness over long time periods [99]. These findings go some way to revealing the important role of non-coding UC sequences in neural development, shedding light, for the first time, on the significant impact of ultraconserved ‘dark matter’ on embryo development.

## Transcribed UCEs

Several studies demonstrated that a large subset of UCEs is actively transcribed, though without any protein-coding potential [103–106]. These transcripts, known as T-UCEs, act as lncRNAs regulating other RNAs, and include (and often extend beyond) the conserved sequences initially described by Bejerano et al. [89]. Interestingly, members of this new family of transcripts are expressed in a tissue-specific manner and exhibit aberrant expression levels in several human cancers, as reported for the first time by Calin and colleagues [104]. T-UCEs are preferentially located in the cytoplasm, where they are able to influence gene-expression levels and regulate several biological processes, such as cell proliferation and differentiation. The main molecular mechanism of T-UCE activity described to date is its ‘decoy’ function. By acting as natural sponges, these lncRNAs can sequester miRNAs via sequence complementarity [107–110]. Decoy binding sites within T-UCEs also act through chromatin remodeling by interplaying with distinct regulatory molecules including epigenetic modifiers, transcription factors, and catalytic proteins [111, 112]. Although the recent technological advances have revealed the molecular circuitries involving T-UCEs in human diseases and in development, their functional activities still remain largely unexplored. For a functional genome-wide characterization of T-UCEs, precise and rigorous gene annotation would require enhanced bioinformatics and high-throughput RNA sequencing approaches [13]. T-UCEs can in fact be transcribed from either the sense or antisense strand of the encoding gene, corresponding to the sense and complementary sequence, respectively, and are generally referred to as ‘+’ and ‘+A’. To date, a total of 962 T-UCEs have been annotated, further supporting independent transcriptional regulation by neighboring protein-coding genes [89, 113]. Although growing evidence has underscored the importance of T-UCEs during the early stages of development, and in stem cell biology, the physiological role of this specific class of lncRNAs and their mechanism(s) of action is only recently emerging [114, 115].

### Role of T-UCEs during embryonic development

Due to their highly conserved nature, T-UCEs are emerging as new and critical players of key developmental gene regulation orchestrating the patterning of cells into tissues and organs during development [116–118].

**Fig. 3:**
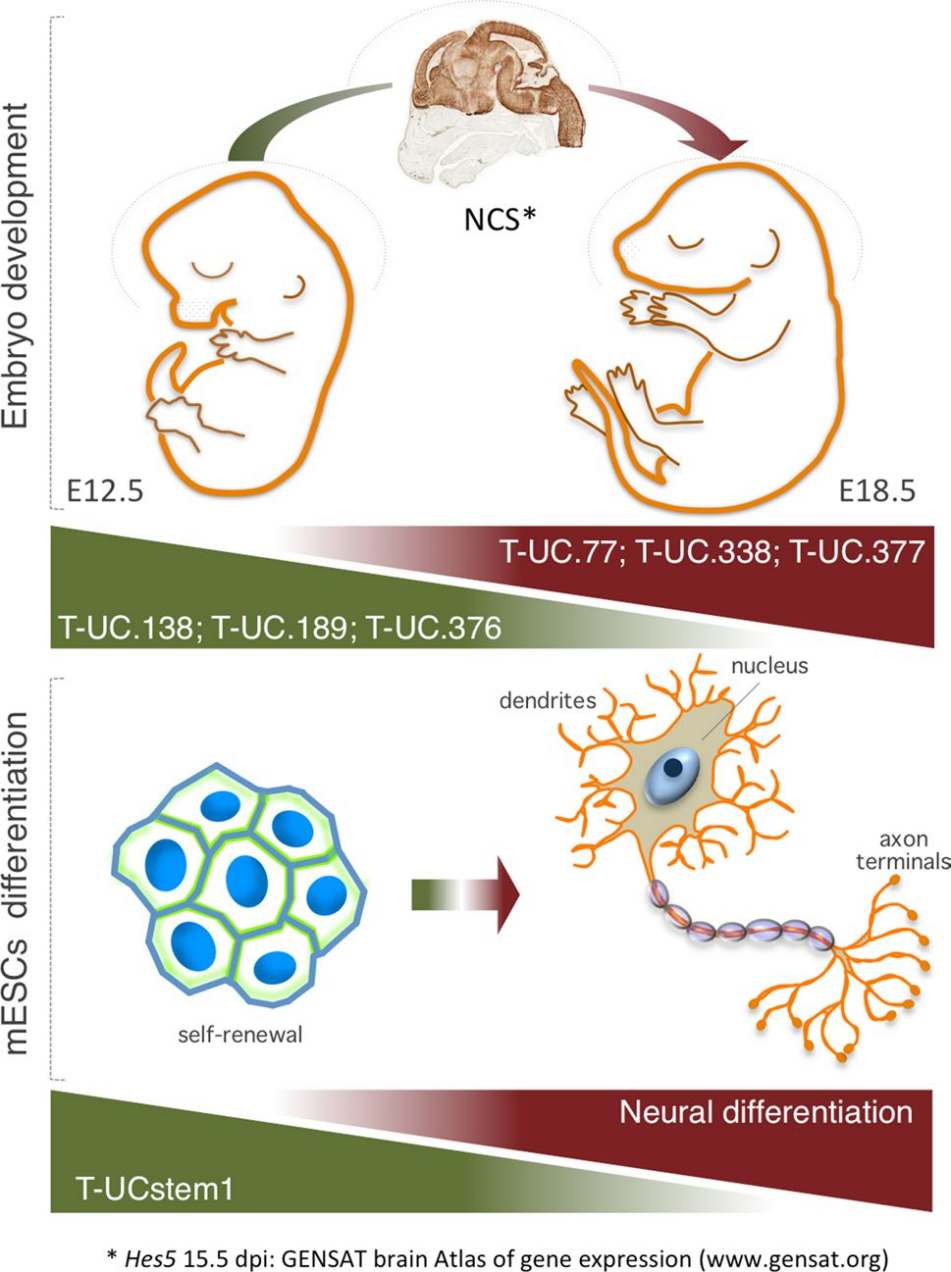
T-UCEs showing dynamic expression profiles during E12.5–E18.5 of mouse brain development (top panel). T-Ucstem1 expression decreases during ESC neural differentiation (bottom panel)

The majority of UCEs are transcribed into single-stranded transcripts during development, and exhibit regional and cell-specific localization. They are involved in the formation of a broad range of cell types, and in ensuring correct embryonic patterns. By combining large-scale genome-wide expression analysis and in situ hybridization detection, several groups showed that T-UCEs are differentially expressed in both time and space with highly restricted expression in selected regions of the mammalian embryo [117–119]. High-resolution screening of RNA sequence signals derived from annotated ultraconserved sequences revealed that 76 UCEs are actively transcribed and specifically enriched in the nervous system of developing brain at embryonic day E14.5 [119]. In particular, T-UC.77, T-UC.338, T-UC.377, and T-UC.359 show dynamic expression profiles increasing during E12.5–E18.5 of mouse brain development. In contrast, T-UC.138, T-UC.189, and T-UC.376 display decreasing expression patterns in the same time frame, indicating physiological significance in earlier stages of mouse brain development [117] (Fig. 3, top panel). Of note, some T-UCEs remain expressed in adult brain, functioning in homeostasis in the cerebral cortex [119].

Intriguingly, most T-UCEs also retain high conservation of gene expression among different species. Indeed, a large spectrum of T-UCEs exhibit a similar expression pattern and cell-specific localization during development across mice, macaques, and humans, indicating that DNA sequence conservation may also correspond to conserved expression and function during evolution [117]. T-UCEs can also cooperate with TFs, promoting the differential and fine-tuned regulation of nearby genes controlled by the same DNA regulatory elements. One example is the ultraconserved lncRNA *Evf*-*2*, localized downstream of the *Dlx5* locus and transcribed antisense to *Dlx6*, which regulates neural cell fates in the brain. *Evf*-*2* controls GABAergic interneuron activity in *cis* by regulating cellular levels of the TFs Dlx5 and Dlx6, while it inhibits DNA methylation by interacting simultaneously in *trans* with the transcriptional activator Dlx2 and the repressor Mecp2. In particular, *Evf*-*2* prevents site-specific CpG DNA methylation of the Dlx5/6 enhancer by modulating competition between Dlx2 and Mecp2 in the medial ganglionic eminence at E13.5 [120, 121].

### T-UCE-mediated regulation of ESC self-renewal and differentiation

Although the previous findings mostly linked T-UCEs to human diseases such as cancer, more recent evidence provides a new dimension to the understanding of T-UCE functions in regulating basal cellular physiology, demonstrating a direct involvement of T-UCEs in stem cell biology. Due to their polyhedral nature, T-UCEs can interplay with different types of molecular factors, DNA, RNA, and proteins, and this confers the capacity to transduce higher order regulatory networks maintaining the balance between self-renewal and multi-lineage differentiation. T-UCE family members are dynamic and temporally regulated in gene expression during stem cell differentiation [122].

To date, an unbiased genome-wide expression analysis approach has been the starting point for identifying tissue-specific lncRNAs in many studies. Dinger et al. described, for the first time, the developmentally regulated expression of T-UCEs during differentiation of mouse ESCs. By integrating genomic context analysis with expression profiling of transcripts, they identified novel candidates with a potential role in self-renewal and cell fate choice. Among the T-UCEs examined, *Evf*-*1/2* and the antisense *Dlx1as* showed progressively increasing expression levels during cardiovascular differentiation, and exhibited coordinated expression profiles with their respective genomically associated coding genes, *Dlx5*/*Dlx6* and *Dlx1*/*Dlx2* [49]. Notably, *Dlx1as* is expressed in adult mice in brain regions associated with neurogenesis including anterior sub-ventricular zone and olfactory bulb acting as a crucial neural development regulator in the glial–neuronal lineage specification of multipotent adult stem cells [123].

More recent large-scale efforts, employing genome-wide sequencing of multiple tissues, identified T-UC.283+ as the T-UCE most expressed in ESCs, iPSCs and in several embryonic and extra-embryonic tissues. Although no specific function of T-UC283+ has been elucidated, it has been suggested that it may be involved in pluripotency maintenance but also during the first-cell lineage specification [107].

At genomic level, by examining histone methylation in mouse ESCs across 56 large UCE-rich loci, most highly conserved non-coding elements in mammalian genomes were found grouped within regions enriched for key genes of differentiation and developmental patterning. The association of conserved non-coding sequences with ‘bivalent domains’, which represent a chromatin-based mechanism for maintaining pluripotency and silencing developmental genes in mouse ESCs, revealed their potential role in epigenetic regulation. Indeed, most of the ultraconserved DNA sequences contained specific binding sites of SUZ12 enabling trimethylation at histone H3K27 in pluripotent stem cells [124, 125]. These findings highlighted the crucial functions of ultraconserved loci in chromatin state regulation for the correct induction of cell differentiation programming.

**Fig. 4:**
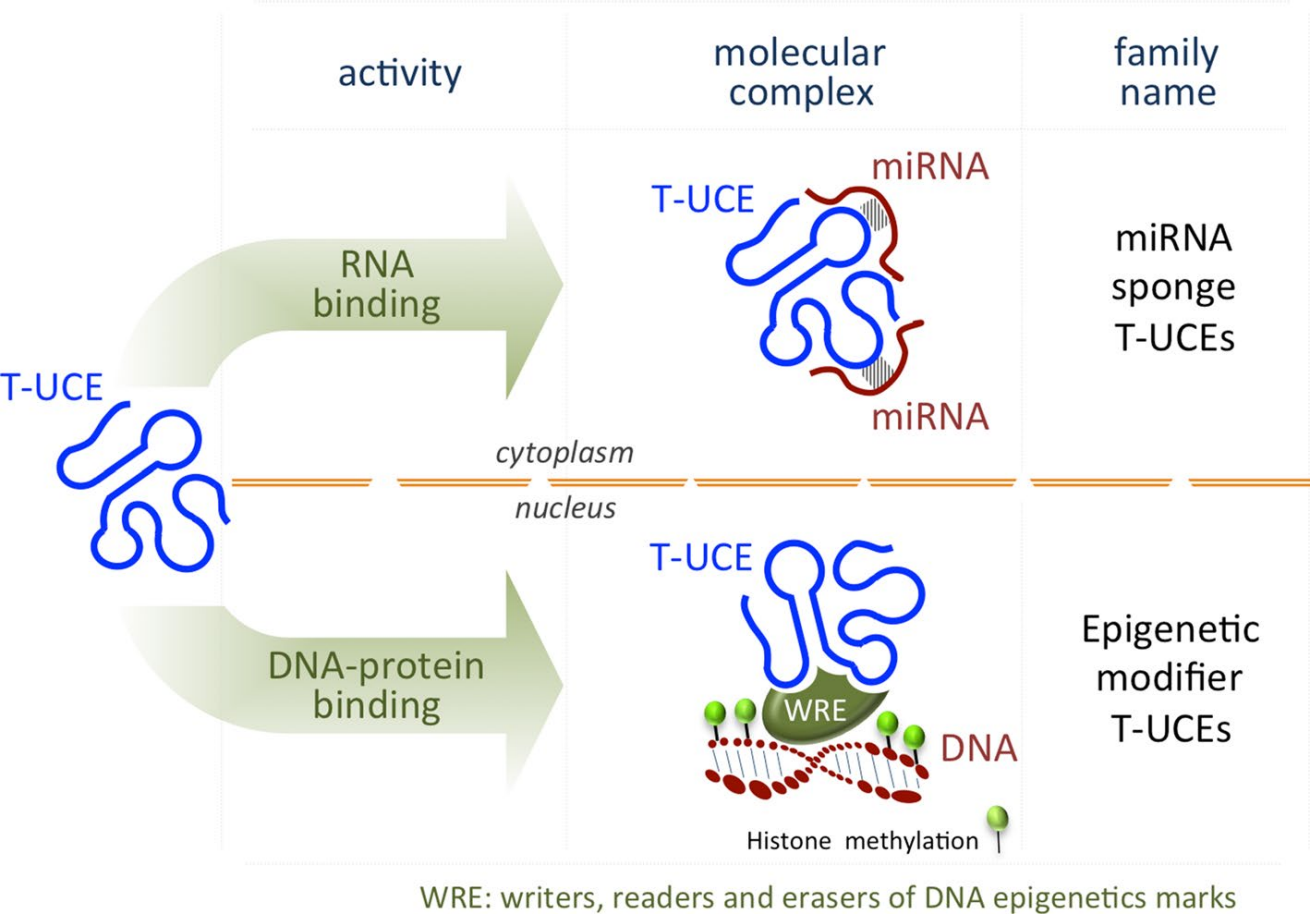
Scheme representing the T-UCE mode of action in ESCs

One of the most functionally characterized T-UCEs in stem cell biology is *T*-*UCstem1*, which fine-tunes the balance between ESC self-renewal and differentiation (Fig. 3, bottom panel) by exerting a dual but distinct role in the nucleus and in the cytosol (Fig. 4). Nuclear *T*-*UCstem1* directly interacts with PRC2, facilitating its recruitment to chromatin on bivalent domain-associated genes. Indeed, in the absence of *T*-*UCstem1*, PRC2 is displaced resulting in an increased H3K4me3/H3K27me3 ratio at bivalent domains, driving ESCs to rapidly exit from pluripotency and undergo differentiation. In the cytosol, *T*-*UCstem1* controls cell cycle progression in ESCs by acting as a sponge, sequestering miR-9, and de-repressing its mRNA targets *TLx1* and *Lin28* [126] (Fig. 4).

Given the well-known function of miR-9 in the embryonic and post-natal neurogenesis [127], it will be interesting to investigate in the future whether T-UCstem1 may exert a regulatory role also in adult neurogenesis, particularly in the neural stem cells of the sub-ventricular zone.

Together, all these studies explore the role of conserved lncRNAs in stem cell biology and provide a better understanding of their modes of action within an already complex regulatory landscape, which may be useful in developing more effective stem cell-based clinical strategies (Table 3).

**Table 3:** UCE and T-UCE

	Biological context	Function	References
UCE			
UC.248, 329, 463, 465, 467, 470, 482	Brain development	Enhancer activity	[98, 99]
T-UCE			
*Evf*-*1/2*	ESC-cardiac differentiation (EBs)	Chromatin modification	[49]
*Dlx1as*	Differentiation	Chromatin modification Competitive endogenous RNA	[123].
*T*-*UC.283*+	Embryonic and extra-embryonic tissues/iPSCs	Not defined	[107]
*T*-*UCstem1*	Undifferentiated ESCs	Competitive endogenous RNA and chromatin modification	[126]
*T*-*UC.77, 338, 377, 359,* 138, 189, 376	Brain development	Not defined	[117]

## Conclusions and future perspectives

Despite the increasing interest, our understanding of the biological functions and molecular mechanism of lncRNAs is still in its infancy. Major challenges towards this goal are the lack of functional annotation for the majority of lncRNAs and low intracellular expression levels. In addition, their mostly weak sequence conservation precludes the use and comparison of vertebrate model organisms (e.g., zebrafish, mouse, and human) for studying fundamental biological processes including stem cell differentiation and embryonic development. This has prompted scientists to focus on a specific subgroup of conserved/ultraconserved lncRNAs, such as T-UCEs, which show a high level of interspecies conservation to decipher their molecular basis.

Despite extensive knowledge of lncRNAs biology, their applications in diagnosis and therapy still require further investigations, including better elucidation of their mode of action. For instance, given their cell-type- and organ-specific expression patterns, lncRNAs might be employed as potential selection markers for screening suitable stem cells/iPSCs or progenitor cells [128]. In this context, gaining a greater insight into ultraconserved lncRNAs may help frame and guide further studies into less conserved classes of lncRNAs.
